# Manual Fractional Technology with CO_2_ Laser Combined with Transdermal Drug Delivery for Hypertrophic Scar: A Retrospective Study

**DOI:** 10.1007/s00266-025-04837-5

**Published:** 2025-04-02

**Authors:** Houhuang Qiu, Bingliang Wu, Fuqiang Pan, Siyuan Zhou, Liming Zhang, Xiang Zhou

**Affiliations:** 1 Department of Medical Cosmetology, Second Affiliated Hospital of Guangxi Medical University, No. 166 Daxue East Road, Nanning, 530000 Guangxi China; 2 Guangxi Medical University, No. 6 Shuangyong Road, Nanning, 530000 Guangxi China

**Keywords:** Hypertrophic scar, Manual fractional technology with CO_2_ laser, Laser-assisted drug delivery, Triamcinolone acetonide, 5-fluorouracil

## Abstract

**Background:**

Hypertrophic scars are a common fibrotic skin disorder with a high recurrence rate. While various treatment options exist, their efficacy remains uncertain. Manual fractional technology with CO_2_ laser technology, a novel approach, has shown promise; however, its effectiveness as a standalone ablative treatment is limited. This retrospective study aims to evaluate the clinical outcomes of combining Manual fractional technology with CO_2_ laser technology with triamcinolone acetonide and 5-fluorouracil transdermal delivery for the treatment of hypertrophic scars.

**Objective:**

This study retrospectively evaluated the clinical efficacy of MFT with CO_2_ laser combined with transdermal triamcinolone acetonide and 5-fluorouracil in treating hypertrophic scars.

**Methods:**

A cohort of 42 patients with 48 hypertrophic scars underwent the combination therapy thrice. Scar evaluations were conducted using the patient and observer scar assessment scale (POSAS) before treatment and one month after each session. Adverse and complication reactions were monitored, and patients were followed for 6 months post-treatment. Hypertrophic scar recurrence and patient satisfaction were assessed.

**Results:**

The total POSAS score significantly decreased from pre-treatment [48.00 (43.00, 53.75) VS 21.29±7.167; *p*<0.001]. In the subgroup analysis, treatment outcomes varied significantly by skin type, with scar quantity and genetic factors influencing remission time. The recurrence rate during the 6-month follow-up was 6.24%, with a satisfaction rate of 83.34%. No severe adverse reactions were reported.

**Conclusions:**

The findings indicate that laser-assisted drug delivery using MFT with CO_2_ laser demonstrates significant clinical efficacy, a low recurrence rate, and an absence of serious adverse reactions in treating hypertrophic scars. This method shows promise as a novel treatment modality for hypertrophic scars.

**Level of Evidence IV:**

This journal requires that authors assign a level of evidence to each article. For a full description of these Evidence-Based Medicine ratings, please refer to the Table of Contents or the online Instructions to Authors  www.springer.com/00266.

## Introduction

Annually, millions of patients globally develop scars due to various causes, with hypertrophic scars occurring in 40–94% of postoperative patients and 30–91% of burn patients [1, 2]. It is characterized by the excessive proliferation of fibroblasts and vessels, along with pathological deposition of extracellular matrix (ECM) components. Beyond causing itching, pain, and alterations in appearance, this condition negatively impacts both physical and mental health. Currently, numerous clinical treatment modalities exist, including pharmacotherapy, laser therapy, surgery, physical compression therapy, radiation therapy, and cryotherapy. However, monotherapies often carry a higher risk of recurrence and demonstrate variable efficacy. A universally accepted gold standard treatment regimen remains elusive. Consequently, clinicians are increasingly focusing on combination therapies, which can more effectively mitigate recurrence rates by leveraging complementary mechanisms [3].

Laser-assisted drug delivery (LADD), a combination therapy, has demonstrated significant promise in treating hypertrophic scars. The fundamental principle involves utilizing the microscopic thermal zone (MTZ) created by fractional laser to transdermally deliver drugs to deep lesions, thereby exerting therapeutic effects [4]. However, the limited treatment depth of fractional laser renders it less effective for thick scar tissues. To address this, Lei et al. [5] introduced "Manual Fractional Technology (MFT)." In this modality, the operator determines the surface area, depth, and density of each MTZ based on the local thickness and severity of the pathological scars. Additionally, proliferated vessels around the periphery and superficial middle areas of pathological scars can be occluded to reduce blood supply and release the contracture bands formed by hypertrophic scars. Regarding pharmacotherapy, triamcinolone acetonide combined with 5-fluorouracil (5-FU) is widely utilized. These drugs inhibit fibroblast proliferation by suppressing TGF-β_1_ expression and angiogenesis by disrupting vascular endothelial growth factor (VEGF) signaling [6]. Laser-assisted delivery of corticosteroids or 5-FU for pathological scar treatment has demonstrated clear therapeutic efficacy, all of which are based on the fractional CO_2_ laser-based LADD technique [7–11]. Currently, no reports exist on the efficacy of LADD based on MFT with CO_2_ laser for hypertrophic scars. This study thus aimed to evaluate the efficacy and safety of MFT with CO_2_ laser combined with transdermal triamcinolone acetonide and 5-FU in treating hypertrophic scars, providing new insights into hypertrophic scar treatment.

## Materials and Methods

This retrospective observational study, adhering to the Declaration of Helsinki principles, received approval from the Ethics Committee of the Second Affiliated Hospital of Guangxi Medical University [Approval No. 2023-KY (0774)]. The study utilized clinical data devoid of patient identification information, with written informed consent obtained from all participants prior to treatment.

### Eligibility Criteria

Inclusion criteria:

1. Diagnosis of hypertrophic scar by two plastic surgeons based on established criteria [12], with the presence of one or more hypertrophic scars;
2. Patients aged 18–60 years, providing written informed consent;
3. Undergoing treatment with MFT using CO_2_ laser combined with transdermal triamcinolone acetonide and 5-FU.

Exclusion criteria:

1. Pregnant or lactating women;
2. Dysfunction of hepatorenal or other vital organs, bone marrow suppression, mental illness, or other serious conditions;
3. Allergy to triamcinolone acetonide and 5-FU;
4. Recent hypertrophic scar treatments within the past 6 months;
5. Treatment frequency less than three sessions;
6. Poor compliance with follow-up.

### Patient Data

From December 2016 to January 2024, fifty-eight patients fulfilling the inclusion criteria who received treatment at the Medical Cosmetology Department of the Second Affiliated Hospital of Guangxi Medical University were enrolled. Exclusions comprised fourteen patients with fewer than three treatments and two patients subjected to multiple treatment modalities during the observation period. Ultimately, 42 patients were included in the study.

### Source of Drugs and Laser Equipment

The study utilized fluorouracil injection (10 ml: 0.25g, molecular weight 130.08Da, t_1/2_ 10–20 minutes, Shanghai Xudong Haipu Pharmaceutical Co., Ltd.), triamcinolone acetonide acetate injection (1 ml: 40 mg, molecular weight 476.54Da, t_1/2_ 5 hours, Zhejiang Xianju Pharmaceutical Co., Ltd.), sodium chloride injection (10 ml: 0.09 g, molecular weight 58.44Da, China Otsuka Pharmaceutical Co., Ltd.), and JY laser SPLB-200A (Chongqing Jingyu Laser Technology Co., Ltd.).

### Treatment

Prepare a mixed solution with normal saline as solvent, 5-FU 4mg/mL + triamcinolone acetonide 10mg/mL [6, 13, 14]. Anesthetic cream was applied to the lesion for one hour. Subsequently, MFT with a CO_2_ laser (Incision handpiece, focal length: 100 mm, energy density: 40–60J/cm^2^, pulse width: 1–3 ms, frequency: 40–60Hz, spot size: 0.5 mm, spot spacing: 3–5mm) was performed. Post-surgery, the mixture was immediately applied to the wound, allowing for spontaneous absorption over 5–10 minutes, repeated three times. Chlortetracycline hydrochloride was applied to the wound post-treatment until healed. A total of three treatments were administered; Laser therapy was performed by the same plastic surgeon.

### Outcome Evaluation

The primary outcome measure was the patient and observer scar assessment scale (POSAS). The patient scale was self-assessed by the patient before treatment and one month after each treatment session. The observer scale was assessed from photographs by two plastic surgeons not involved in the treatment, with their average score recorded.

Secondary outcome measures included recurrence, adverse reactions, complication and patient satisfaction, gathered retrospectively through online surveys during treatment and six months post-treatment.

### Data Analysis

Statistical analysis was performed using SPSS 27.0. The Kolmogorov–Smirnov test assessed data normality. For normally distributed measurement data, results were expressed as x±s and analyzed using the t test; nonnormally distributed data were represented as M (P_25_, P_75_) and analyzed using the Wilcoxon rank sum test. Enumeration data were analyzed with the chi square test or Fisher's exact probability test, while ranked data were evaluated using the Wilcoxon rank sum test. Kaplan–Meier survival analysis was employed to assess the influence of subgroup factors on time to remission. A *p* value below 0.05 denoted statistical significance.

## Results

### Patient Characteristics

A retrospective study included 42 patients (20 males, 22 females). Among them, four had two hypertrophic scars, and one had three, totaling 48 hypertrophic scars. The median age (P_25_, P_75_) was 25.50 years (20.00, 31.25); the median disease duration (P_25_, P_75_) was 5.50 months (3.00, 27.00). The primary causes were trauma (52.38%) and surgery (28.58%), with the face (64.58%) and neck (18.75%) being the most affected sites (Table 1).

**Table 1:** General characteristics of patients (N)

Item	Male	Female
N, %	20(47.62%)	22(52.38%)
Number of hypertrophic scars (%)	21(43.75%)	27(56.25%)
Age (years)	28.77 $\pm$ 10.109	24.60 $\pm$ 6.311
Disease duration (months)	4.00(2.75,24.00)	7.50(3.25,45.00)
*Cause* (%)		
Trauma	14(33.33%)	8(19.05%)
Surgery	2(4.76%)	10(23.82%)
Burns	0	3(7.14%)
Skin infectious diseases	3(7.14%)	1(2.38%)
Tattoos	1(2.38%)	0
*Site* (%)		
Face	16(33.33%)	15(31.25%)
Neck	2(4.17%)	7(14.58%)
Chest and back	0	2(4.17%)
Upper extremity	3(6.25%)	0
Lower extremity	0	3(6.25%)

### Efficacy Evaluation

**Fig. 1:**
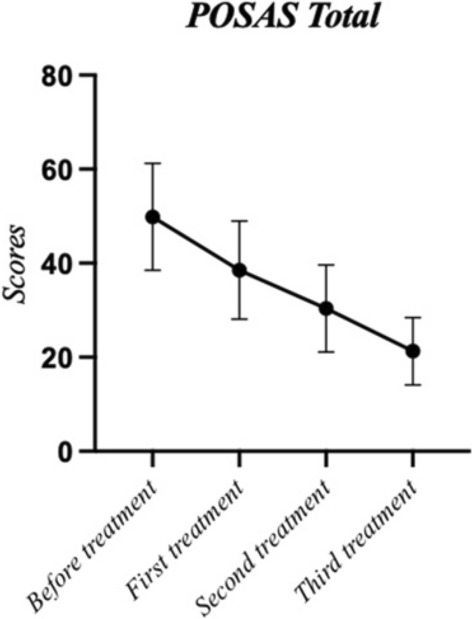
POSAS score. Significant differences in POSAS scores were observed before treatment and at each subsequent treatment point (*p*<0.05)

The primary outcome measure, POSAS score, showed substantial improvement after the third treatment compared to pre-treatment levels (*p*<0.001), with a significant downward trend observed between treatments (Fig. 1). Both the observer and patient assessment scores indicated significant differences post-third treatment (*p*<0.001, *p*<0.001). All POSAS subitems—vascularity, pigmentation, thickness, pliability, surface area (observer scale), and pain, itching, color, stiffness, thickness, and regularity (patient scale)—demonstrated marked improvement following the third treatment (*p*<0.001) (Table 2). In the analysis of the influence of various subgroup factors on treatment outcomes, no significant intra-group differences were observed for gender, duration, scar quantity, and genetic factors (*p*>0.05). However, in the skin type subgroup, significant differences in treatment efficacy were observed among the three skin types (*p*<0.001). Specifically, type II exhibited significantly better outcomes compared to type III and type IV (*p*<0.001, *p*<0.001), while the treatment effects of type III and type IV were comparable, with no significant difference between them (*p*>0.05) (Table 3).

**Table 2:** Comparison of POSAS scores pre-treatment and post-third treatment [(Mean ± SD), M (P_25_, P_75_)]

Score	Before treatment	After the third treatment	*p*
Total	48.00(43.00,53.75)	21.29 $\pm$ 7.167	$<$ 0.001
Observer Scale	22.50(20.00,24.75)	10.50(6.25,12.75)	$<$ 0.001
Vascularity	4.00(3.00,5.00)	2.00(1.00,2.00)	$<$ 0.001
Pigmentation	5.00(5.00,7.00)	2.00(1.25,3.00)	$<$ 0.001
Thickness	3.00(3.00,4.00)	1.00(1.00,2.00)	$<$ 0.001
Pliability	6.00(5.00,7.00)	2.00(1.00,3.00)	$<$ 0.001
Surface Area	3.00(3.00,4.00)	2.00(2.00,3.00)	$<$ 0.001
Patient Scale	25.00(23.25,29.75)	11.00(8.00,13.75)	$<$ 0.001
Pain	2.00(2.00,3.00)	1.00(1.00,1.00)	$<$ 0.001
Itching	5.13 $\pm$ 2.049	1.00(1.00,2.00)	$<$ 0.001
Color	6.00(5.00,7.00)	3.00(2.00,3.00)	$<$ 0.001
Stiffness	6.00(5.00,6.00)	2.00(1.00,2.00)	$<$ 0.001
Thickness	3.00(3.00,4.00)	2.00(1.00,2.00)	$<$ 0.001
Regularity	4.00(3.00,5.00)	2.00(2.00,3.00)	$<$ 0.001

**Table 3:** Subgroup analysis of MFT with CO_2_ laser treatment for hypertrophic scars based on POSAS score [(Mean ± SD), M (P25, P75)]

Subgroup	Sample size (*n*)	Before treatment	After the third treatment	Difference (Pre - Post)	*p*
*Gender*					
Male	21	49.00(46.00,50.00)	23.62 $\pm$ 5.749	25.05 $\pm$ 7.978	0.051
Female	27	47.00(41.00,58.00)	16.00(13.00,26.00)	31.30 $\pm$ 12.443	
*Skin Type*					
Type II	8	61.875 $\pm$ 15.950	14.00(12.25,25.50)	44.375 $\pm$ 11.747	$<$ 0.001
Type III	28	47.464 $\pm$ 9.954	21.286 $\pm$ 7.378	26.179 $\pm$ 8.410	
Type IV	12	47.417 $\pm$ 4.738	23.833 $\pm$ 6.379	23.583 $\pm 6.230$	
*Duration*(months)					
$<$ 6	23	51.130 $\pm$ 12.706	20.826 $\pm$ 7.947	30.304 $\pm$ 11.275	0.185
6-12	9	49.00(45.50,58.50)	22.00(19.00,25.00)	31.444 $\pm$ 12.561	
$>$ 12	16	46.125 $\pm$ 6.141	21.688 $\pm$ 7.236	24.438 $\pm$ 9.252	
*Scar quantity*					
Single	37	47.182 $\pm$ 9.745	14.818 $\pm$ 4.665	32.364 $\pm$ 9.003	0.198
Multiple	11	48.00(43.00,53.50)	23.216 $\pm$ 6.663	27.432 $\pm$ 11.478	
*Genetic factor*(n)					
Positive	9	48.000 $\pm$ 3.606	24.556 $\pm$ 6.167	23.444 $\pm$ 6.839	0.125
Negative	39	48.00(42.00,57.00)	20.539 $\pm$ 7.240	29.745 $\pm$ 11.580	

**Fig. 2:**
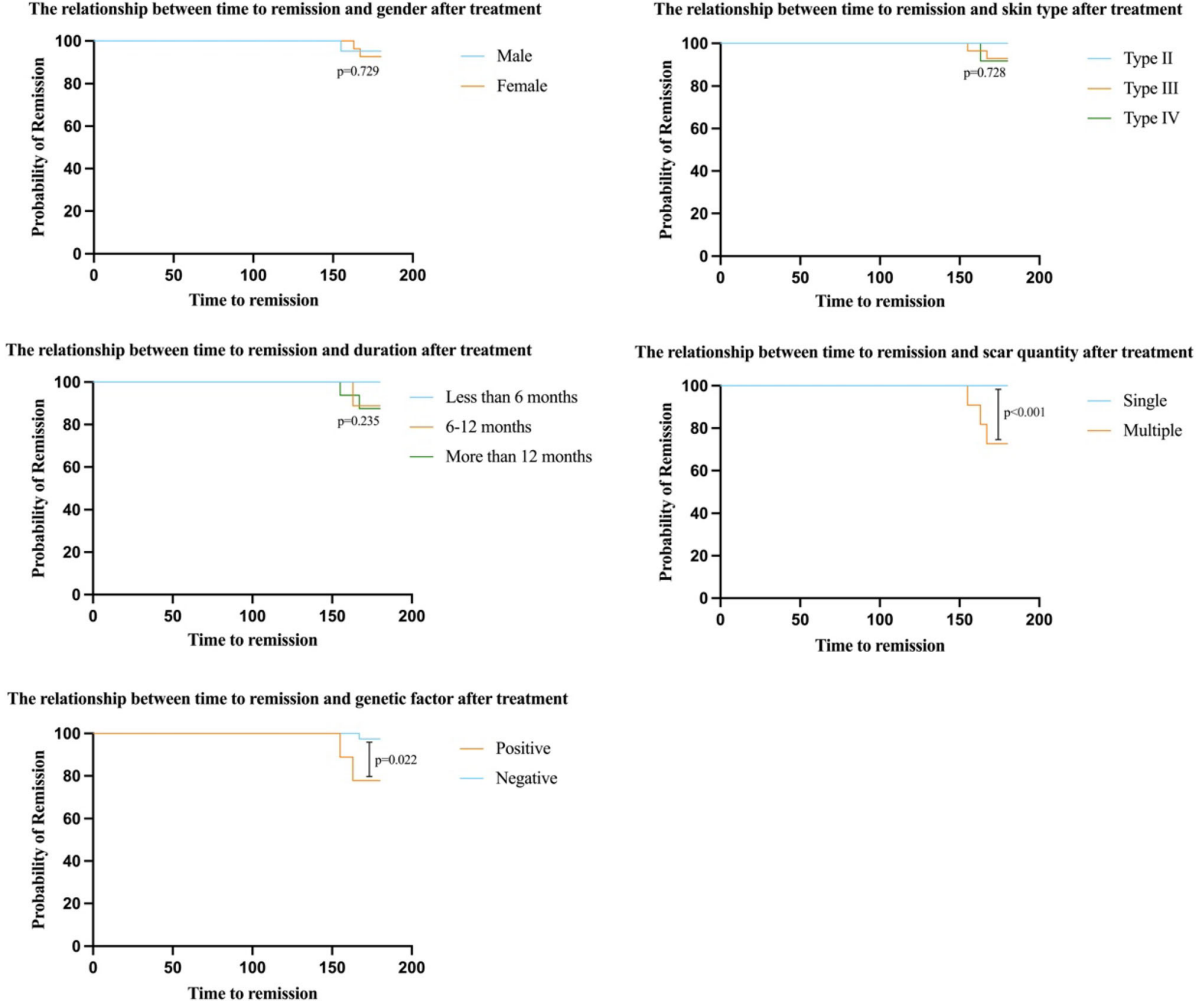
Analysis of the impact of various subgroup factors on the time to remission after treatment. Gender, skin type, and duration did not significantly influence the time to remission following treatment. In the subgroup analysis based on scar quantity, patients with a single scar demonstrated a significantly longer time to remission compared to those with multiple scars. In the subgroup analysis related to genetic history, patients without a genetic history of hypertrophic scars experienced a significantly longer remission time compared to those with a positive genetic history

Secondary outcome measures encompassed recurrence rate, adverse reactions, complication and patient satisfaction. Recurrence was defined by the emergence of one or more conditions within the 6-month follow-up after the final treatment: enlarged hypertrophic scar, vascular proliferation, congestion and redness, itching, and/or pain symptoms. During follow-up, three cases of recurrence (6.24%) were reported: one female experienced an enlarged hypertrophic scar, and two patients reported itching symptoms in the lesion area. The mean recurrence time was 5.4 months (Table 4). In the analysis of subgroup factors affecting post-treatment remission time, no significant differences were found for gender, skin type, or duration (*p*>0.05). However, patients with a single scar had longer remission times than those with multiple scars (*p*<0.05). Additionally, patients with a positive genetic history were more prone to recurrence than those with a negative history (*p*<0.05) (Fig. 2).

**Table 4:** Adverse reactions, recurrence, complication and satisfaction by sex (N)

Item		Male	Female	*p*
*Adverse reactions* (%)				
Transient redness and swelling		21(40.38%)	27(51.92%)	1.000
Transient pigmentation		2(3.85%)	2(3.85%)	
*Complication* (%)				
Functional impairment	Pre-treatment	2(4.76%)	1(2.38%)	0.071
	Post-third treatment	1(2.38%)	0	
Psychological negative impact	Pre-treatment	3(7.14%)	8(19.05%)	0.014
	Post-third treatment	0	3(7.14%)	
Infection		0	0	
Malignant transformation		0	0	
*Recurrence* (%)				
Recurrence		1(2.08%)	2(4.16%)	1.000
No recurrence		20(41.68%)	25(52.08%)	
*Satisfaction* (%)				
Very satisfied		10(23.81%)	14(33.34%)	0.622
Satisfied		6(14.29%)	5(11.90%)	
Neutral		3(7.14%)	1(2.38%)	
Dissatisfied		1(2.38%)	2(4.76%)

Adverse reactions were predominantly characterized by transient redness, swelling, and pigmentation. Post-treatment, all patients exhibited temporary redness and swelling at the treatment site, which resolved spontaneously within 1–3 days. Four patients experienced transient pigmentation, occurring after the second treatment (*n* = 1) or the final treatment (*n* = 3), with a mean resolution time of 2.5 months. No other adverse reactions, including poor wound healing or depigmentation, were noted during follow-up. Additionally, adverse reactions did not significantly differ by sex. Common complications of hypertrophic scars, aside from itching and pain, include functional impairment, infection, malignant transformation, and psychological distress. Three patients (7.14%) had functional limitations, particularly in elbow and knee joint mobility. Additionally, 11 patients (26.19%) experienced appearance-related anxiety. After three treatment sessions, one patient (2.38%) still showed mild elbow joint restriction, but this change was not statistically significant (*p*>0.05). Of the 11 patients with anxiety, 3 (7.14%) did not improve. However, the reduction in psychological distress, especially anxiety related to appearance, was statistically significant (*p*<0.05) (Table 4).

Patient satisfaction was categorized as very satisfied (*n* = 24, 57.15%), satisfied (*n* = 11, 26.19%), neutral (*n* = 4, 9.52%), and dissatisfied (*n* = 3, 7.14%). The overall satisfaction rate reached 83.34%, with no significant sex differences observed (Table 4).

## Discussion

**Fig. 3:**
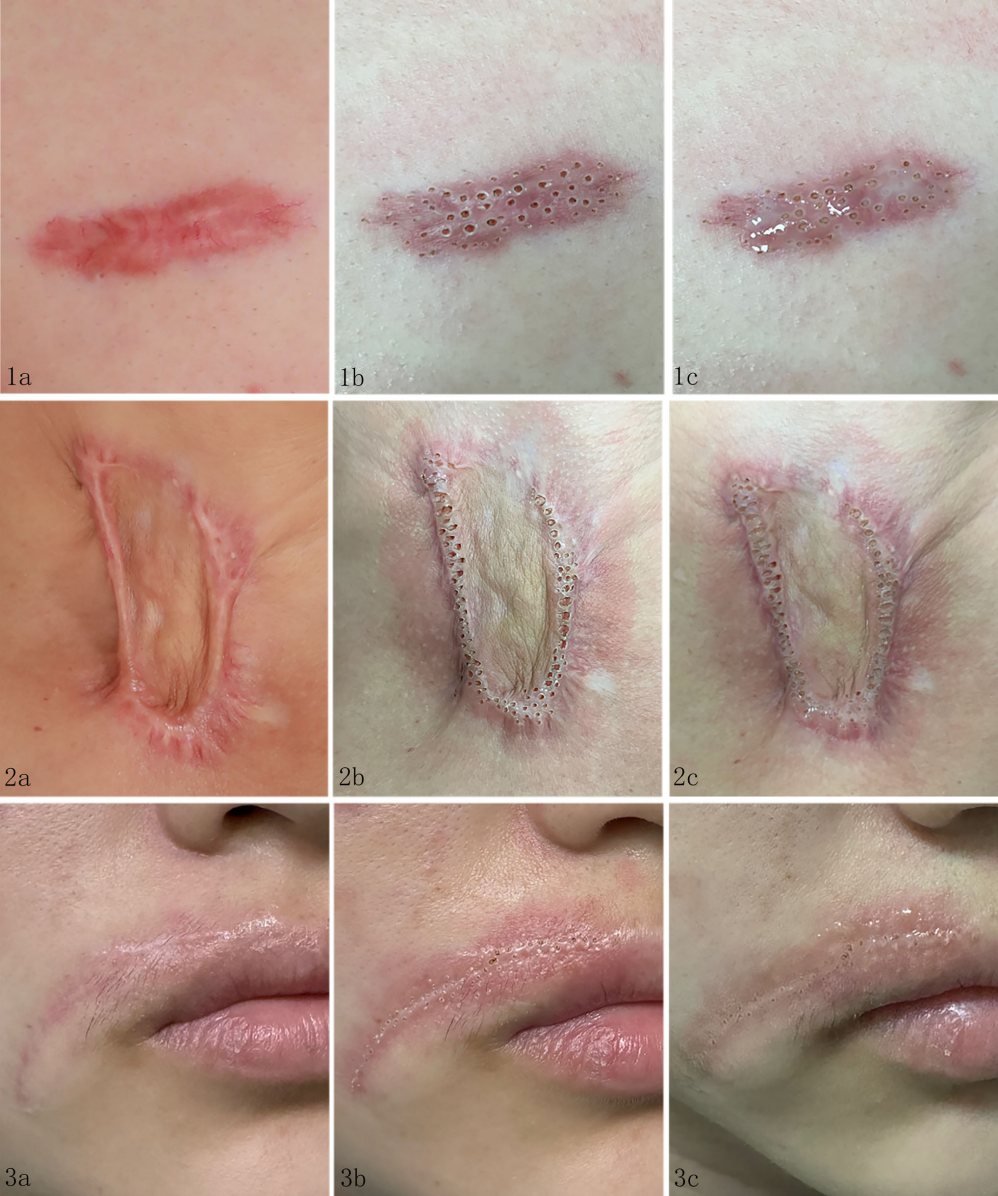
Details of MFT with CO2 laser combined with transdermal triamcinolone acetonide and 5-FU delivery. 1a. Scar tissue exhibits numerous abnormally proliferating capillaries with an uneven surface. 1b. MFT with CO2 laser occludes along the vascular contour; the MTZ formed in thicker areas is deeper with a larger surface area. Capillaries disappear immediately post-treatment. 1c. Apply the mixed medical solution for spontaneous absorption. 2a. Scar tissue displays many contracture bands of varying local thicknesses. 2b. The density of the MTZ formed by MFT with CO2 laser at the scar contracture band increases; deeper MTZ formation in thicker areas with larger surface area. Normal tissues around the scar are released immediately post-treatment. 2c. Apply the mixed medical solution for spontaneous absorption. 3a. Scar tissue is thin and locally uneven. 3b. Compared to other cases, the MTZ formed by MFT with CO2 laser is shallower and has a reduced surface area and density. 3c. Apply the mixed medical solution for spontaneous absorption

The fractional CO_2_ laser plays a therapeutic role on the principles of “fractional photothermolysis theory” [15]. This technique creates MTZs, stimulating skin repair mechanisms, thus fostering regeneration. MFT with CO_2_ laser represents an advancement over traditional fractional CO_2_ lasers. In this study, spot spacing was meticulously controlled at 3–5 mm to prevent superimposed thermal effects and excessive thermal injury, thereby enabling surrounding normal tissues to aid in wound repair and promote healing. The fractional CO_2_ laser’s ability to enhance topical drug efficacy by promoting penetration is well-documented [11, 14, 16]. MTZs facilitate the delivery of both lipophilic and hydrophilic drugs with molecular weights below 500 Da [17]. The triamcinolone acetonide acetate and fluorouracil injections utilized in this study conformed to this molecular weight criterion. No chemical reactions between these drugs have been reported, and the pore size of the fractional CO_2_ laser typically measures around 0.5 mm. In this study, MTZs created by MFT with CO_2_ laser ranged from 0.5 to 2.0 mm, significantly larger than those formed by standard fractional CO_2_ lasers, thereby enhancing drug penetration and absorption. Complete spontaneous absorption of the drug mixture occurred within 5–10 minutes post-application. The half-lives of both drugs exceeded 10 minutes, ensuring sustained therapeutic concentrations during tissue penetration and utilization (Fig. 3).

Ablative CO_2_ laser therapy alone exhibits a higher recurrence rate in the treatment of hypertrophic scars, with uncertain efficacy [18]. As a prevalent combination therapy, LADD not only establishes a drug delivery channel but also synergizes laser and pharmacotherapy for enhanced outcomes. While MFT effectively disrupts fibroblasts, it inevitably induces inflammation during wound repair. Multiple inflammatory cytokines, including TGF-β1, can stimulate fibroblast regeneration and abnormal collagen secretion [19]. The combination therapy of triamcinolone acetonide and 5-FU, utilizing MTZ, targets scar tissues at all layers, directly inhibiting abnormal fibroblast proliferation and collagen deposition during wound repair, mitigating the inflammatory response, and suppressing VEGF expression to reduce neovascularization. Patients may experience intolerable severe pain during drug injections, hindering the completion of necessary treatments. LADD offers a significant advantage by reducing treatment-related pain and enhancing patient compliance [7].

**Fig. 4:**
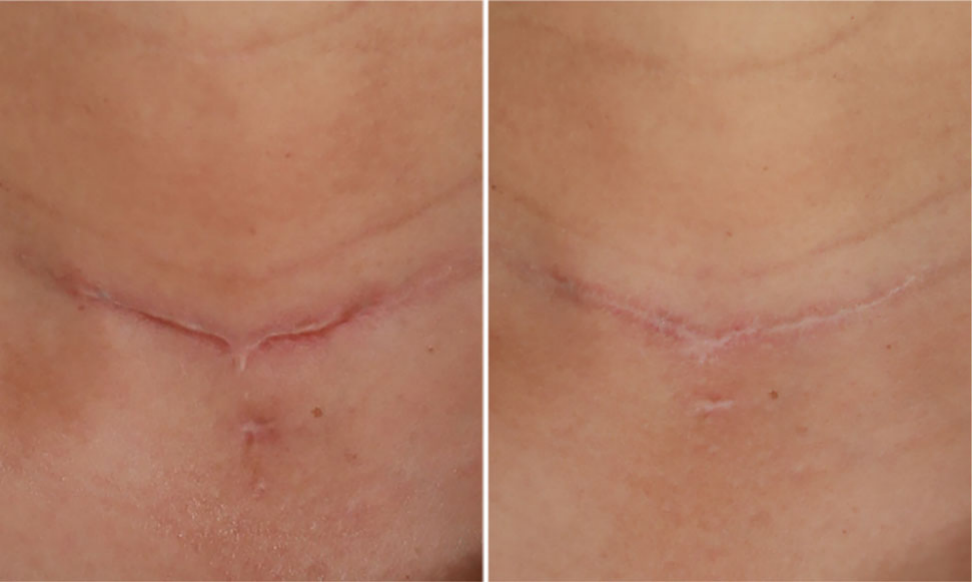
A 56-year-old female with a hypertrophic scar on her neck due to thyroid surgery (4 months). Pre-treatment (left); post-third treatment (right)

**Fig. 5:**
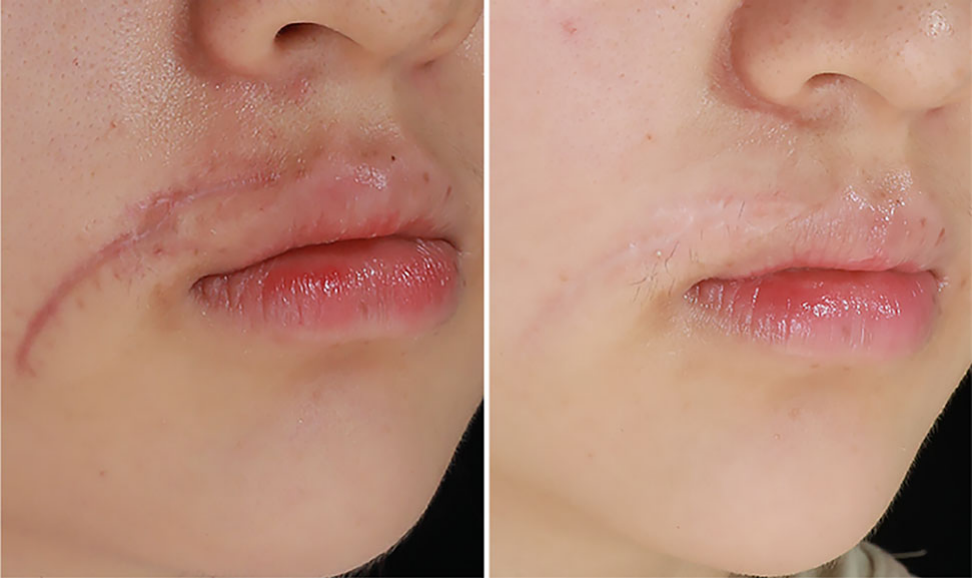
A 26-year-old female with a hypertrophic scar on her face from trauma (1 month). Pre-treatment (left); post-third treatment (right)

**Fig. 6:**
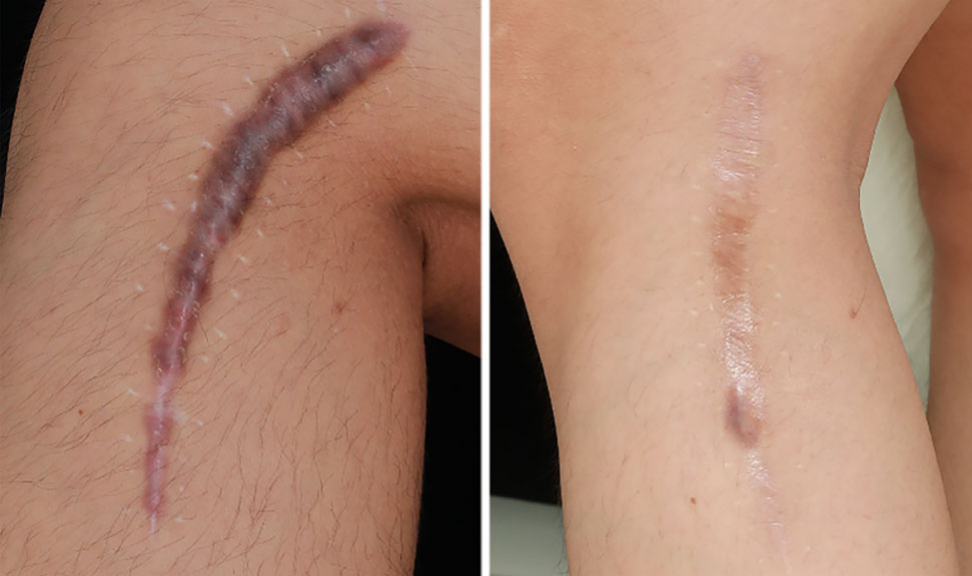
A 24-year-old female with a hypertrophic scar on her left lower leg (6 months) post-fracture surgery. Pre-treatment (left); post-third treatment (right)

This retrospective study highlights the superior efficacy of this treatment modality. Post three treatments, significant improvements were observed in the thickness and color of scar tissues, as well as in itching and pain symptoms. An increasing number of treatments correlated with a progressive decline in the total POSAS score. Under the same treatment protocol, lighter skin types show better outcomes, while results in Type III and IV skin are similar. Maninder et al. [20] further corroborate this perspective in their study. Patients with a positive genetic history and multiple hypertrophic scars had a significantly shorter time to remission. Although the genetic predisposition in patients with multiple scars remains unconfirmed, a positive family history may be a key risk factor. Hypertrophic scars typically exhibit rapid growth during the first 3 to 6 months, transitioning to a quiescent phase before maturing over approximately 2 years [21]. The 6-month follow-up revealed a recurrence rate of 6.24%, covering the proliferative phase when recurrence risk is highest. Compared to recurrence rates of 9–50% with corticosteroid injections [22] and 19–47% with 5-FU monotherapy [23], this suggests the combined treatment may reduce recurrence. Further randomized controlled trials are required in the future to confirm these results. Shin et al. [24] employed combination therapy with a non-ablative fractional laser and intralesional triamcinolone injection for pathologic scars, resulting in a remission period of 4.17 months. In contrast, this study achieved a remission period of 5.4 months, demonstrating a significant delay in scar recurrence. Typical cases were depicted in Figs. 4, 5, 6.

Waibel et al. [7] investigated the efficacy of fractional CO_2_ laser combined with triamcinolone acetonide versus 5-FU for hypertrophic scar treatment. Both approaches demonstrated significant efficacy; however, triamcinolone acetonide treatment was associated with corticosteroid-related complications. This study utilized a drug combination to maximize therapeutic benefits while minimizing adverse reactions. The established methods aimed to optimize efficacy with minimal adverse effects. To our knowledge, notable complications of corticosteroid and 5-FU injections include dermal atrophy, telangiectasia, and injection site ulcers [22, 23], while laser therapy commonly results in hypertrophic scarring, pigmentation, and depigmentation [25]. In this study, except for transient pigmentation, no other listed adverse reactions occurred during follow-up. Transient pigmentation resolved spontaneously with treatments such as enhanced physical sun protection and avoidance of cosmetic irritation, with a duration not exceeding three months. All patients experienced transient redness and swelling post-treatment, lasting 1–3 days, likely due to laser therapy. Routine application of chlortetracycline eye ointment facilitated wound protection, improving symptoms without exacerbation. The treatment interval for some patients was 1–2 months, with no observed scar hyperplasia, thus shortening the treatment course and preventing adverse reactions from repeated laser therapy within a short period. This approach ensured efficacy and improved patient compliance.

MFT with CO_2_ laser combined with transdermal delivery of triamcinolone acetonide and 5-FU offers advantages such as convenience, high efficiency, minimal invasiveness, and superior clinical outcomes. Patient satisfaction rates could achieve 83.34%. For patients concerned about the significant trauma and high costs associated with traditional surgical resection, this combined treatment modality offers a more cost-effective and widely accessible alternative, aligning with the growing preference for minimally invasive or non-invasive procedures in modern surgical practice.

This study indicates that MFT with CO_2_ laser combined with transdermal triamcinolone acetonide and 5-FU is effective. As there is currently limited literature on MFT with CO_2_ laser, further clinical studies are necessary to establish standardized protocols. In this study, we have retrospectively reviewed and summarized the treatment parameters employed (Table 5). However, further optimization of drug concentration, laser parameters, and other specifics is necessary. Conducted as a retrospective study with a limited sample size, some patients experienced varied treatment intervals, introducing potential limitations. Future randomized controlled trials will incorporate different drug dosages or laser parameters to identify the optimal treatment regimen. Increasing the sample size and employing more objective parameters will ensure the accuracy and clinical applicability of the findings. A 6-month follow-up period sufficiently encompasses the rapid proliferative phase of hypertrophic scar formation. However, further investigation is necessary to evaluate long-term complications and recurrence rates.

**Table 5:** Recommended treatment parameters for MFT with CO_2_ laser

Scar thickness (mm)	Diameter of MTZ (mm)	Depth of MTZ (mm)	Spacing of MTZ (mm)
<1	0.5–1	<0.5	0.5–2
1–3	0.5–1.5	0.5–1	1–2
3–5	1–3	1–1.5	2–4
>5	2–6	1.5–2.5	4–6

## Conclusion

This study demonstrates that MFT with CO_2_ laser combined with transdermal triamcinolone acetonide and 5-FU is effective, demonstrating a low recurrence rate and absence of serious adverse reactions in treating hypertrophic scars. As an innovative combination therapy, it is straightforward to administer, improves patient compliance, and holds promise as a treatment regimen.
